# Descending thoracic aorta to bilateral femoral artery bypass and thoracic endovascular aortic repair in the management of atypical aortoiliac occlusive disease

**DOI:** 10.1016/j.jvscit.2021.09.010

**Published:** 2021-10-01

**Authors:** Wei Li, Dixon Santana, Elizabeth Rivas, Jonathan Huynh, Roderick Olivas, Amy Edwards

**Affiliations:** a Department of Surgery, Texas Tech Health Sciences Center, Lubbock, Tex; b Department of Anesthesiology, Texas Tech Health Sciences Center, Lubbock, Tex; c Surgical Services, University Medical Center, Lubbock, Tex

**Keywords:** Aortoiliac occlusive disease, Combined repair, Distal thoracic–bifemoral bypass, Intraoperative transesophageal echocardiography, Thoracic endovascular aortic repair

## Abstract

Despite recent advancements in endovascular technology and the proven durability of open surgery, extensive thoracoabdominal aortoiliac occlusive disease (AIOD) remains challenging to treat. In the present report, we have described the case of a 58-year-old woman with AIOD and multiple medical comorbidities. She successfully underwent a novel intraoperative transesophageal echocardiography-guided combined treatment with concurrent descending thoracic aorta to bilateral femoral artery bypass and thoracic endovascular aortic repair. We have shown that this approach, which combines descending thoracic aorta to bilateral femoral artery bypass with thoracic endovascular aortic repair, is an effective treatment alternative for future cases of complex AIOD.

Aortoiliac occlusive disease (AIOD) typically originates at the aortic bifurcation and common iliac arteries, progressing proximally and distally.^1^^,^^2^ Although AIOD can extend to the renal or mesenteric arteries, to the best of our knowledge, no concurrent extensive thoracoabdominal AIOD cases with total occlusion of the distal aorta and bilateral common iliac arteries and severe stenosis in the mid-thoracic aorta have been previously reported.

Percutaneous treatment is now routinely applied to long-segment occlusions such as TASC II D (Trans-Atlantic Inter-Society Consensus class D) lesions.^3^^,^^4^ Nevertheless, open bypass remains a critical part of management when extensive disease or complex anatomy impedes suitable endovascular approaches.^3^^,^^5^, ^6^, ^7^ Additionally, the challenges associated with suprarenal clamping on the mid-visceral atherosclerotic aorta, the presence of a hostile abdomen, or the simultaneous need to correct inflow and outflow have made isolated endovascular and open aortobifemoral bypass methods less attractive.^8^, ^9^, ^10^, ^11^, ^12^ In the present report, we have described a more complex situation that required a procedure to improve inflow proximal to the descending thoracic aorta to bilateral femoral artery bypass (DTFB). The institutional review board provided an exemption, and the patient provided written informed consent for the report of her case details and imaging studies.

## Case report

**Fig 1:**
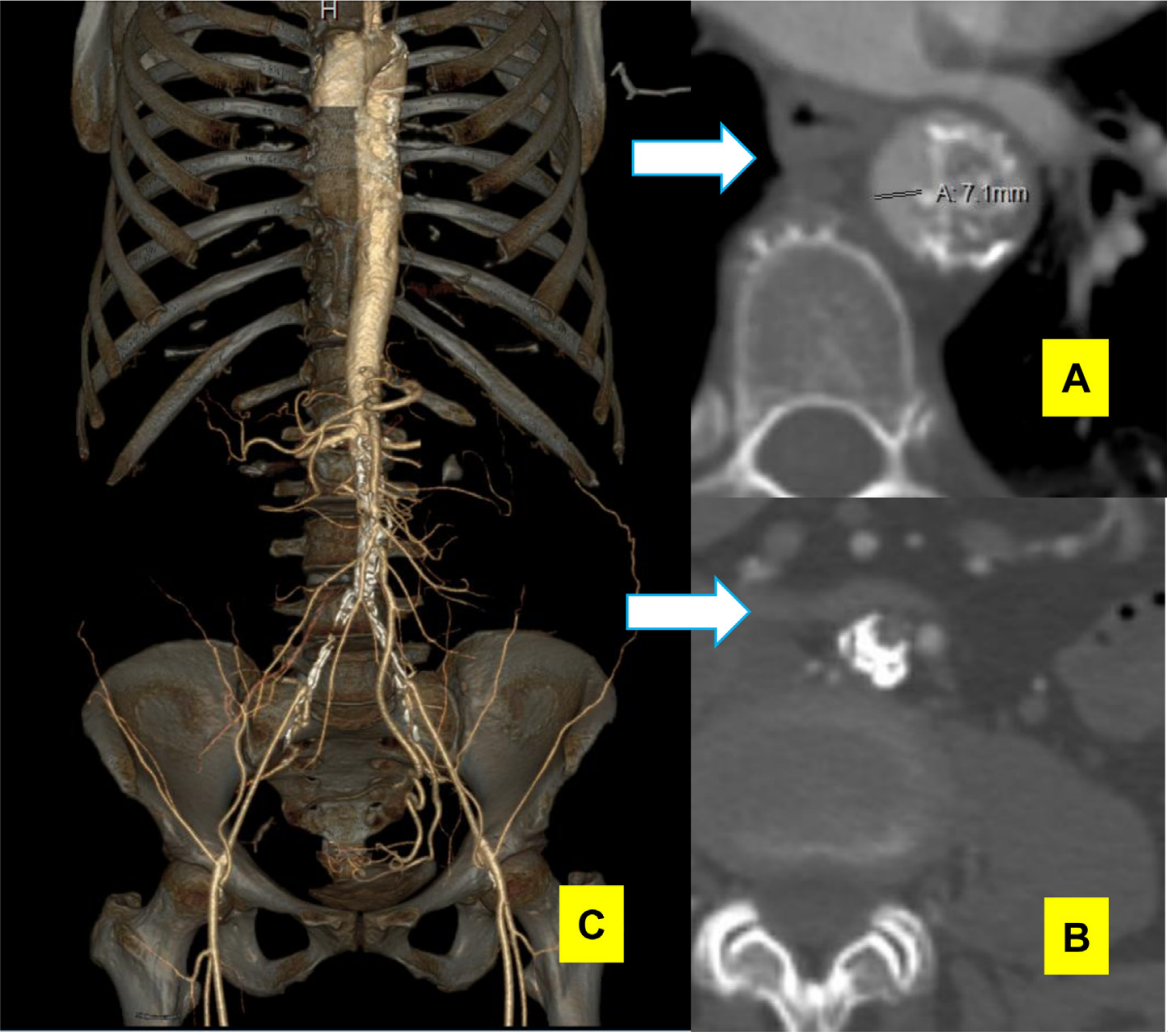
Computed tomography angiogram (CTA) showing severely stenotic aortic segment **(A)**, total occlusion of the distal abdominal aorta **(B)**, and mid-visceral aortic atherosclerosis **(C)**.

A 58-year-old woman with hypertension, coronary artery disease, and chronic obstructive pulmonary disease and surgical history of appendectomy, cholecystectomy, and hysterectomy had been transferred from another hospital's vascular surgery service because of short-distance claudication and pain at rest. A chest computed tomography angiogram (CTA) revealed a stenotic segment (diameter, 7.1 mm; Fig 1, *A*) in the proximal descending thoracic aorta. The examination demonstrated absent bilateral femoral, popliteal, and pedal pulses with monophasic signals throughout, consistent with a recent ankle brachial index of 0.4 bilaterally. The CTA also demonstrated additional mid-visceral aortic severe atherosclerosis and heavily calcified total occlusion of the distal infrarenal abdominal aorta and bilateral common iliac arteries, with reconstitution of the external iliac arteries (Fig 1). The remaining distal arterial vasculature contained gross atherosclerotic disease but remained patent with inline flow to the feet. Cardiology and anesthesia considered the patient to have reasonable physiologic tolerance for major surgery with expected recovery. In line with the patient's desire, we proceeded with a combined approach.

**Fig 2:**
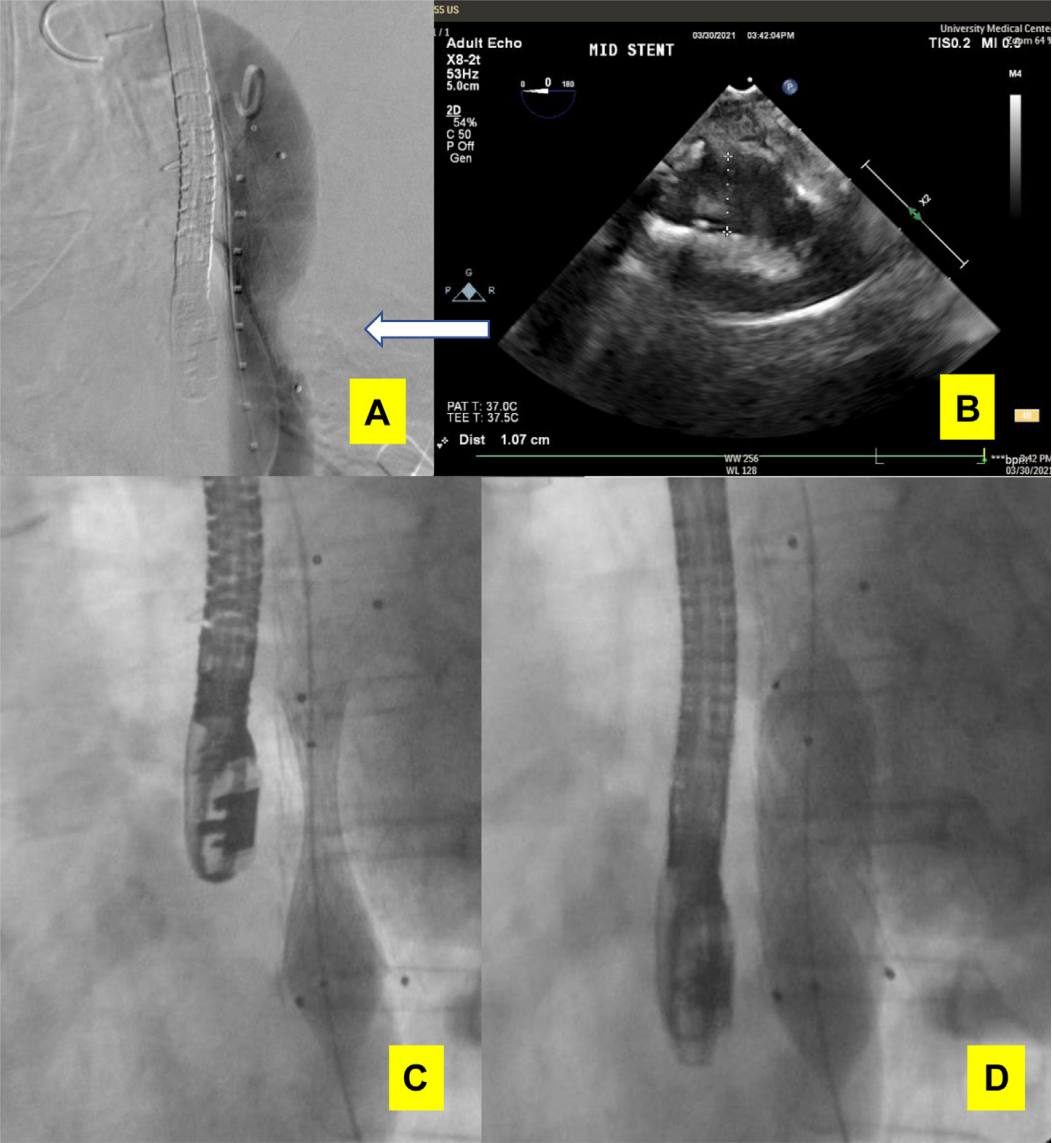
Intraoperative simultaneous fluoroscopy and live transesophageal echocardiography (TEE) monitoring during Endurant-II stent-graft (Medtronic) and Palmaz stent (Cordis) placement.

**Fig 3:**
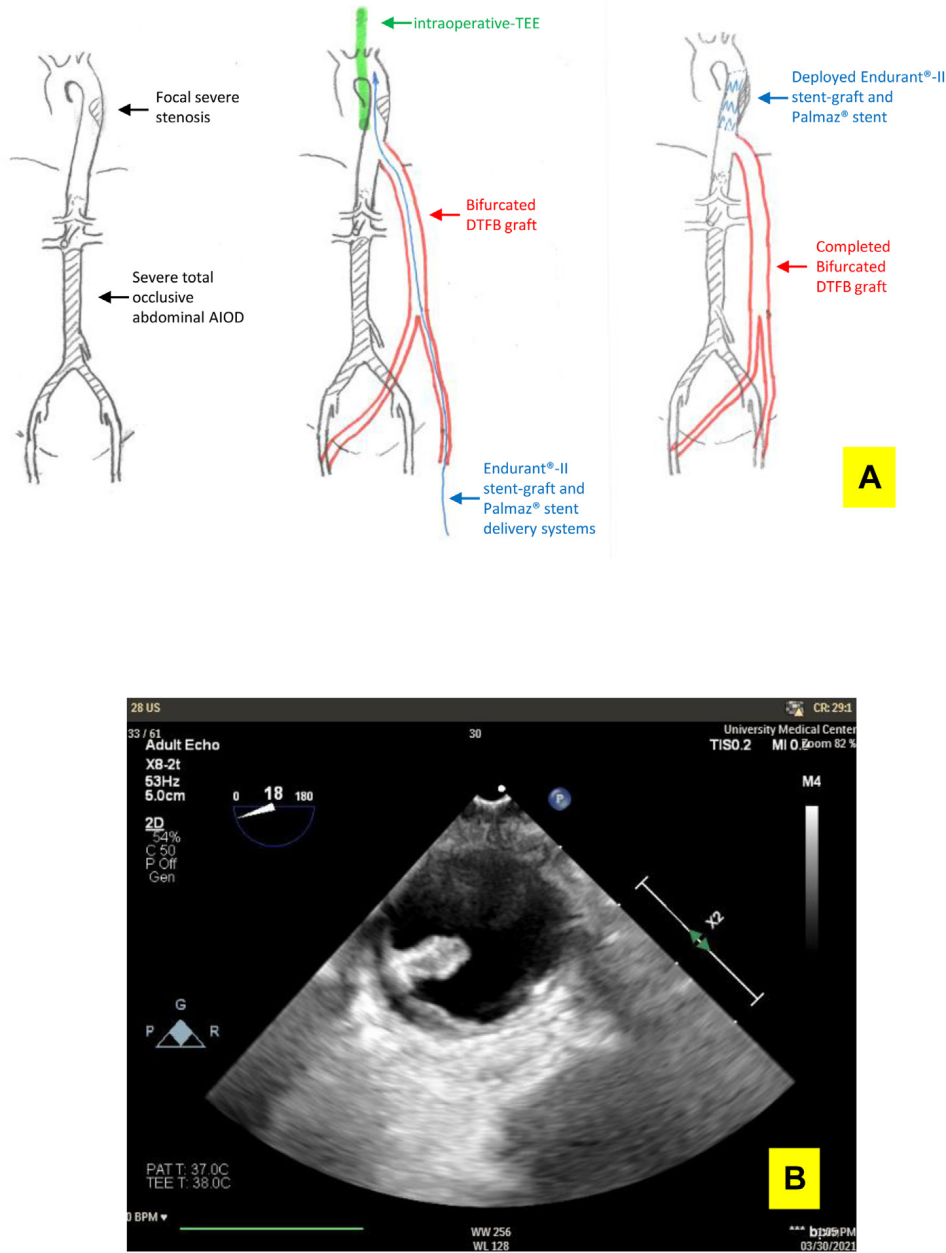
**A**, Diagram of the surgery. **B,** Intraoperative transesophageal echocardiography (TEE) finding of an unstable plaque within the planned distal clamping area for proximal anastomosis. *AIOD,* Aortoiliac occlusive disease; *DTFB,* descending thoracic aorta to bilateral femoral artery bypass.

Modified from previous DTFBs,^9^, ^10^, ^11^, ^12^ blunt dissection for graft tunneling anterior to the external iliac vessel facilitated entry into the left lateral retroperitoneal space. Simultaneously, we created a preperitoneal tunnel behind the rectus muscles from left to right, from above to below the groin. After exposure of the distal descending aorta through a left posterolateral thoracotomy, an intraoperative transesophageal echocardiography (TEE) probe was advanced to precisely define the severely stenotic segment of the thoracic aorta. TEE confirmed that a segment of the distal thoracic aorta was the only zone suitable for the bypass graft's proximal anastomosis. A retroperitoneal tunnel from the left pleural cavity to the suprainguinal preperitoneal space was created with blunt finger dissection posteromedially to the spleen, posterior to the left kidney, and anterior to the psoas muscle. A bifurcated 16-mm × 8-mm × 8-mm Gelweave graft (Terumo Medical Corp, Somerset, NJ) was tunneled from the distal thoracic aorta to the bilateral femoral arteries. After systemic heparinization, partial side-biting clamping of the distal descending thoracic aorta was achieved during the proximal anastomosis and was continued throughout the case with live TEE monitoring to ensure adequate antegrade flow for visceral perfusion during the end-to-side anastomosis (Figs 2 and 3, *A*).

**Fig 4:**
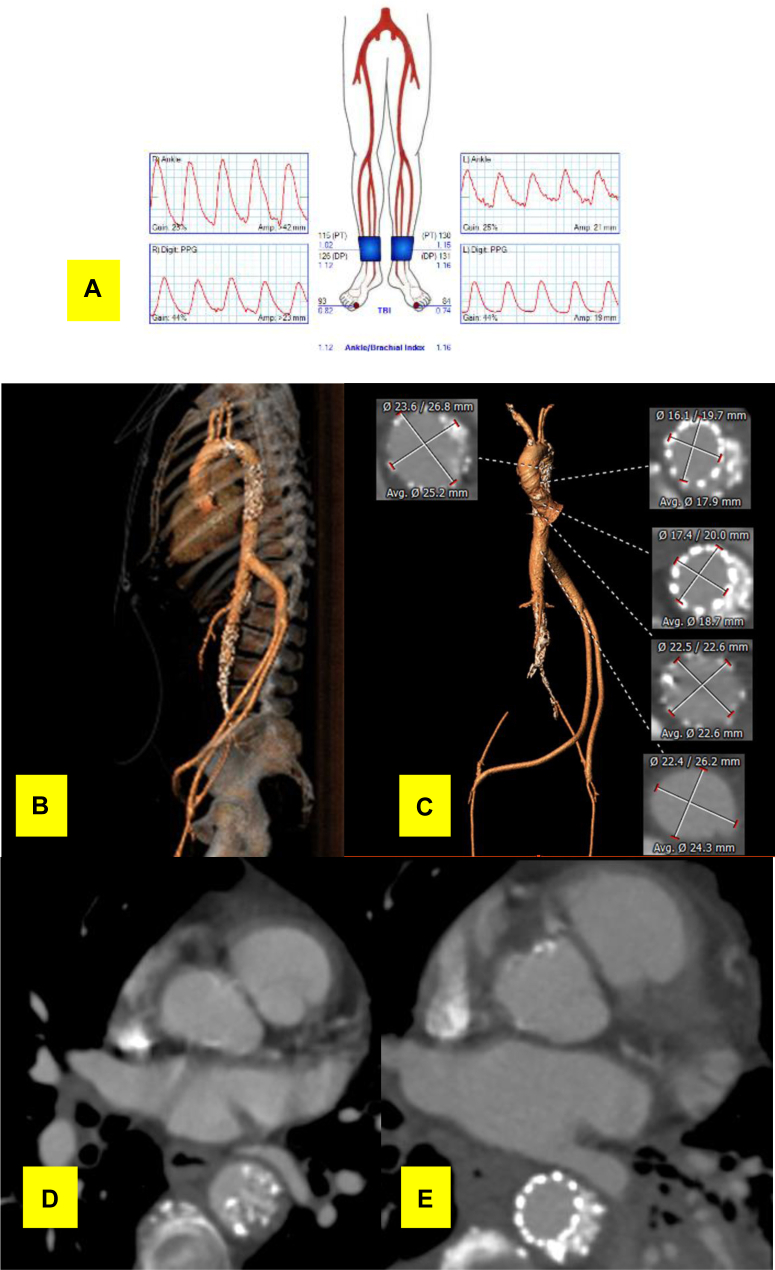
Postoperative follow-up ankle brachial index **(A)**, computed tomography angiogram (CTA; **B and C)**, and the stenotic thoracic aorta pre- **(D)** and postoperatively **(E)**. *Amp.,* Amplitude; *Avg.,* average; *L,* left; *R,* right.

**Fig 5:**
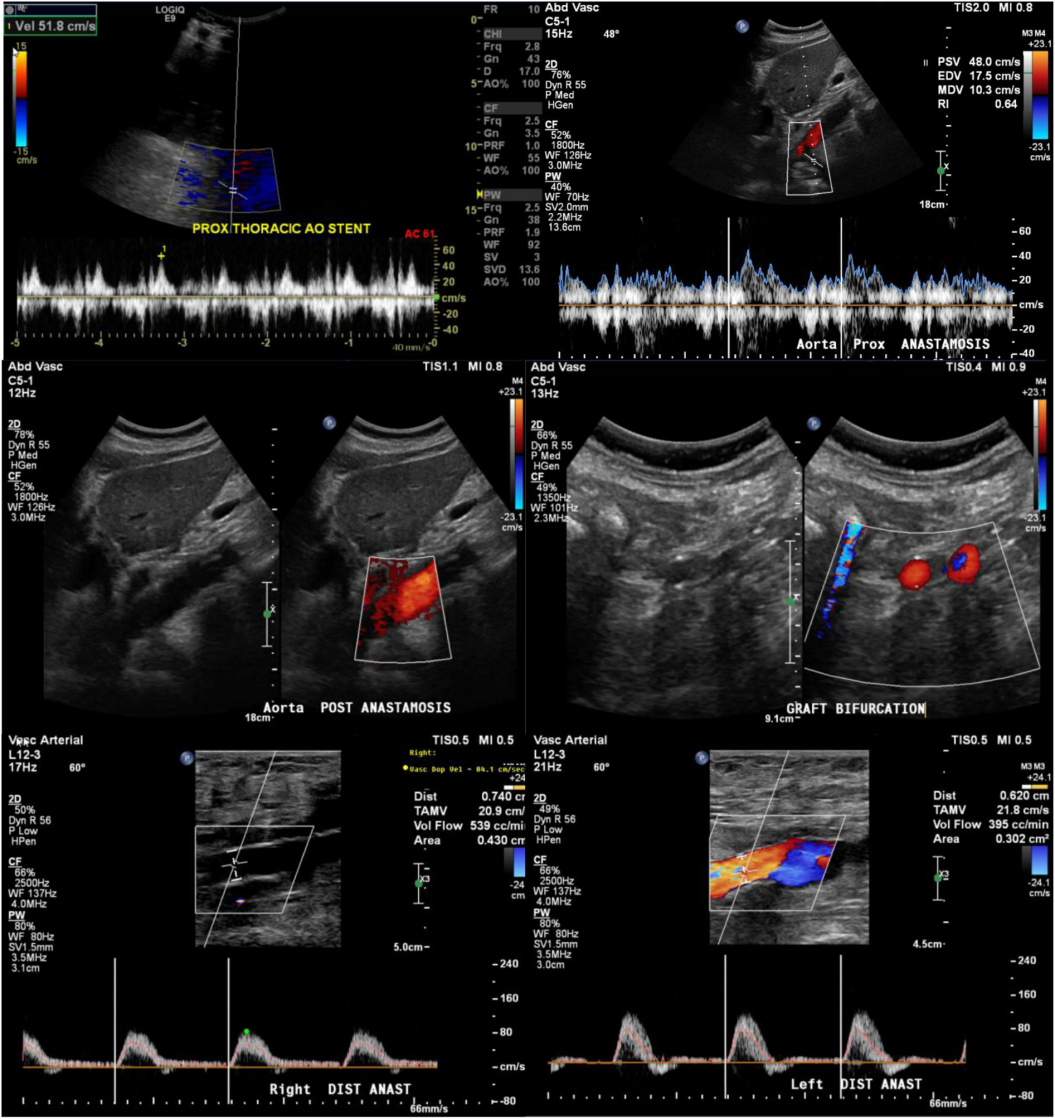
Mid-term (5-month) follow-up duplex ultrasound images showing patent descending thoracic aorta to bilateral femoral artery bypass (DTFB) and thoracic endovascular aortic repair (TEVAR). *ANAST,* anastomosis; *DIST,* distal; *PROX,* proximal.

Using the left-limb 8-mm Gelweave graft as a conduit, an Endurant-II 28-mm × 28-mm × 82-mm iliac extension stent-graft (Medtronic, Minneapolis, Minn) was placed in the severely stenotic segment proximal to the anastomosis (Fig 3, *A*). A follow-up angiogram and live TEE both indicated suboptimal reexpansion of the stenotic preproximal anastomosis aortic segment with only a 1.07-cm diameter (Fig 2, *A* and *B*). Thus, a balloon-expandable Palmaz stent (P5010; 10 mm × 49 mm) mounted on a MAXI LD 20-mm × 40-mm balloon (Cordis, Milpitas, Calif) was deployed within the Endurant-II stent-graft at 4 atm balloon pressure for an expanded stent diameter of 20 mm. We used a manufacturer-suggested technique, which allowed for stent expansion from both ends simultaneously (Fig 2, *C*). Direct fluoroscopy and concurrent live TEE monitoring enabled the expansion and confirmed an expanded lumen diameter and proper stent-graft coverage without atherosclerotic fragments dislodging distally (Fig 2, *C* and *D*). After subsequent angiograms confirmed patency of the visceral vessels, the anastomoses to the bilateral femoral arteries were completed in routine fashion. At 1 week of follow-up, the ankle brachial indexes were normal with patent bypass and thoracic endovascular aortic repair (TEVAR) stent-grafts, visceral perfusion, and reexpansion of the thoracic aortic segment (Fig 4). The patient was discharged to home. At 5 months after surgery, the patient had gradually regained her ability to practice Taekwondo with palpable pedal pulses. A duplex ultrasound study demonstrated consistent graft patency (Fig 5).

## Discussion

DTFB remains reserved for patients with aortic graft failure or graft infection or for whom the transabdominal approach is not feasible.^9^, ^10^, ^11^, ^12^, ^13^, ^14^ DTFB carries a >80% patency rate at 5 years relative to standard aortobifemoral bypass grafting.^9^, ^10^, ^11^, ^12^^,^^14^ Some surgeons have even advocated for DTFB to serve as the primary method for AIOD revascularization because DTFB results in greater and more consistent patency than axillofemoral bypass grafts.^10^, ^11^, ^12^, ^13^, ^14^, ^15^, ^16^, ^17^ In addition, axillofemoral bypass graft could not have amended our patient's very stenotic proximal descending thoracic aorta. Thus, DTFB appeared the most appropriate bypass option for our patient. Compared with the more invasive retroperitoneal tunnels created during DTFB,^18^ the combined retroperitoneal and preperitoneal space tunneling approach minimized the occurrence of postoperative gastrointestinal complications. The bifurcated graft choice also adequately accommodated the concurrent TEVAR.

AIOD involving both thoracoabdominal and iliac vessels is rare. In the present case, intraoperative TEE served a critical role in obtaining a very favorable outcome. Intraoperative TEE has become a safe and invaluable perioperative imaging tool with high sensitivity and specificity during cardiac and aortic trauma surgery.^19^, ^20^, ^21^, ^22^, ^23^ In the present case, TEE served several key roles. First, TEE, along with the preoperative CTA, facilitated the determination of the precise location of the stenotic segment and proximal anastomosis site, which were separated by a very short and limited transition zone length. Using TEE, we found an unstable plaque within the planned clamping area, which was not appreciated on the preoperative CTA. This plaque could have become a source of postoperative distal embolization if it had remained undiscovered (Fig 3, *B*). We, therefore, adjusted the clamping zone and resected the lesion during proximal anastomosis of the bypass graft.

Second, most surgeons have used the side-biting clamp to partially occlude the distal descending thoracic aorta during the proximal end-to-side anastomosis of the DTFB and have used a handheld Doppler probe to confirm distal perfusion.^10^, ^11^, ^12^, ^13^, ^14^, ^15^, ^16^^,^^18^ However, such a method is incapable of providing direct visualization of the residual lumen and an estimation of the volumetric flow to the visceral organs distally. In the present case, the use of intraoperative TEE provided a high precision view of the side-biting partial clamping site within a very tight region and depicted, in real-time, blood flow to the distal organs during the anastomosis.

Third, to restore an adequate lumen with proper radial expansion force, we deployed a balloon-expandable stent within a self-expandable aortic stent-graft. To mitigate the risk of distal embolization during such deployment,^24^^,^^25^ we used real-time TEE monitoring for visualization and sequential (from caudally to cranially) semi-compliant balloon angioplasty of self-expanding stent-grafts to avoid fragments dislodging from the angioplasty during stent and stent-graft placement.

Finally, dynamic intraoperative TEE offered three-dimensional views both before and after stent placement, eliminating the need for multiple two-dimensional angiograms, which minimized the overall use of fluoroscopy and contrast.

## Conclusions

Our patient had presented with a rare form of AIOD that was treated with a novel intraoperative TEE-guided approach that combined DTFB with concurrent TEVAR. Careful preoperative planning with CTA and intraoperative TEE contributed to the success of the present case. We have demonstrated that DTFB with concurrent TEVAR is a viable treatment option for such complex AIOD. Intraoperative TEE-guided advanced endovascular techniques combined with an open strategy could lead to more effective options for future cases of complex AIOD.
